# Electrochemotherapy induces tumor regression and decreases the proliferative index in canine cutaneous squamous cell carcinoma

**DOI:** 10.1038/s41598-019-52461-6

**Published:** 2019-11-01

**Authors:** Denner S. Dos Anjos, Cynthia Bueno, Larissa F. Magalhães, Georgia M. Magalhães, Ewaldo Mattos-Junior, Marcela M. R. Pinto, Andrigo B. De Nardi, Carlos H. M. Brunner, Antonio F. Leis-Filho, Sabryna G. Calazans, Carlos E. Fonseca-Alves

**Affiliations:** 1 Veterinary Science Graduate Program, University of Franca (UNIFRAN), Franca, Brazil; 2 Department of Veterinary Clinic and Surgery, São Paulo State University (UNESP), Jaboticabal, Brazil; 3 Department of Veterinary Pathology, University of Franca (UNIFRAN), Franca, Brazil; 4 Federal Institute of Education, Science and Technology of the South of Minas Gerais - Muzambinho, Minas Gerais, Brazil; 5 Veterinary Pathologist at the CEVEPAT Laboratory, Botucatu, SP Brazil; 6 Department of Veterinary Clinic of University Paulista, São Paulo, Brazil; 7 Department of Veterinary Clinic, School of Veterinary Medicine and Animal Science, São Paulo State University (UNESP), Botucatu, São Paulo Brazil; 8 Institute of Health Sciences, Universidade Paulista – UNIP, Bauru, SP Brazil

**Keywords:** Squamous cell carcinoma, Cancer therapy, Skin cancer

## Abstract

Canine cutaneous squamous cell carcinoma (cSCC) is the most common skin cancer in dogs, and, due to its low metastatic rate, local treatments, such as electrochemotherapy (ECT), promote disease control or even complete remission (CR). This study aimed to evaluate the gene and protein expression of Bcl-2 and Bcl-2 associated X protein (BAX), the proliferative index and clinical parameters in dogs with cSCC subjected to ECT. A prospective nonrandomized clinical study was performed using dogs with naturally occurring cSCC that was treated with ECT. Eighteen lesions from 11 dogs were selected. The tumor size at day 0 (D0) had no impact on survival or prognosis (P > 0.05). Tumor samples had a lower proliferative index after ECT (D21) than before ECT (P = 0.031). The survival of subjects with Ki67 values lower and higher than the Ki67 median value were not significantly different (P > 0.05). Regarding apoptotic markers, there were no significant differences in the gene and protein expression levels of BAX or Bcl-2 at D0 and D21 (P > 0.05) or in the overall survival of subjects with different levels of apoptotic markers. In conclusion, there was no change in BAX or Bcl-2 gene and protein expression in response to ECT at the time points evaluated, but ECT was able to reduce tumor volume and cellular proliferation in cSCC.

## Introduction

Canine cutaneous squamous cell carcinoma (cSCC) is one of the most common skin cancers in dogs from tropical countries, and the most important etiological factor is chronic sunlight exposure^1–3^. However, canine papillomavirus infection, chronic inflammation and immunosuppression might be involved in the development of cSCC^4–7^. The incidence of cSCC depends on the geographic area, as it is less common in countries farther from tropical regions^1^. In humans and dogs, actinic keratosis is an SCC precursor lesion, with more than 80% of human SCC cases derived from previous actinic keratosis^8,9^. Thus, dogs can be considered an animal model of spontaneous cSCC for comparative purposes.

Generally, cSCCs are locally invasive tumors with a low metastatic rate (13%)^10–13^ similar to that in humans (5%); metastasis mainly occurs in locoregional lymph nodes^14–16^. Due to this low risk of metastasis, local treatments, such as surgery, cryotherapy, radiotherapy, photodynamic therapy and electrochemotherapy (ECT), promote disease control and extend survival in most cases^1,17^.

ECT is capable of inducing an inflammatory response, necrosis, scar tissue and apoptosis in tumors^18–20^. Apoptosis is a genetically programmed process for the elimination of damaged cells, and its onset is controlled by numerous interrelated processes that are influenced by extrinsic and intrinsic signals that converge on an effector pathway. Alterations in these pathways are important in the tumorigenesis process, leading to the persistence of neoplastic cells and the promotion of progression and metastasis^21,22^.

Bcl-2 and Bcl-2 associated X protein (BAX) are important proteins in the BCL-2 family, which consists of pro- and antiapoptotic proteins. BAX/Bcl-2 cross-regulation controls apoptosis, cell survival and cell proliferation^23,24^. Several studies have measured the expression of a single apoptosis-associated protein, such as BAX and BCL-2, by immunohistochemistry, flow cytometry or quantitative real-time PCR analyses and correlated its expression with the prognosis of mammary tumors and lymphoma^25–29^. However, conflicting data have been observed among the studies^25–27^. A significant increase in Bcl-2 expression was previously observed in malignant mammary tumors, suggesting the evasion of apoptosis as one step in the progression to metastasis^29^. Bcl-2 and BAX expression was also assessed in another study, with the authors reporting BAX and Bcl-2 overexpression in more aggressive mammary tumors^27^. These authors also suggested that these apoptotic proteins can be accessory parameters for anticipating the biological behavior and prognosis of mammary tumors^27^. Meichner *et al*.^25^ evaluated the Bcl-2/BAX ratio in dogs with lymphoma and identified a higher Bcl-2/BAX ratio in patients with T cell lymphoma than in those with B cell lymphoma. The Bcl-2 protein prevents apoptosis through BAX inhibition, and a high Bcl-2/BAX ratio indicates resistance to apoptosis. As T cell lymphoma is a more aggressive disease, the higher Bcl-2/BAX ratio indicates resistance to apoptosis. In feline cSCCs, neither BAX nor BCL-2 expression was detected. However, in basal cell tumors, BCL-2 expression was higher (23/24) than that of BAX, which was expressed in only seven out of 24 tumors. For the tumors that expressed both BAX and BCL-2, the BAX:BCL-2 ratio was low^30^.

Another important factor to consider when evaluating cSCC is cellular proliferation, which is measured by the levels of proliferative markers. Among the proliferative markers, Ki67 is one of the most utilized in studies^31–34^. During cellular proliferation, the Ki67 protein is expressed during different phases of the cell cycle, including G1, S, G2 and M phases^31–34^. Thus, it is highly sensitive in identifying proliferating cells. Elevated Ki67 expression has been correlated with high proliferative rates, metastatic disease, low disease-free interval, and low overall survival in both dogs and humans with several types of tumors, such as mammary tumors, mast cell tumors, perianal tumors, oral tumors and SCC^31–39^. Overall, high Ki67 expression is indicative of a high proliferation rate, which can counteract the effectiveness of chemotherapeutic agents^31–34^.

ECT has gained popularity in recent years in both human and veterinary medicine^40,41^. ECT is a combination of chemotherapy and localized delivery of electric pulses to the tumor nodule. ECT aims to increase antineoplastic drug diffusion into the cell after cell membrane electroporation, thereby increasing drug cytotoxicity^17^. The use of electroporation has been limited to the delivery of bleomycin or cisplatin^41^, with very few investigations using doxorubicin, and has mostly been confined to the evaluation of antitumor effects in murine models^42–47^. Previously, electroporation has been shown to deliver doxorubicin more efficiently through the cell membrane. Additionally, doxorubicin can act in a specific manner inside cells and is also a substrate for P-gp expression, which is what differentiates this from bleomycin^48–50^. There was previous *in vitro* and *in vivo* evidence of the effectiveness of doxorubicin when administered with electroporation^44,46,47,51^; However, no studies have investigated the effect of doxorubicin associated with electroporation in clinical trials involving dogs.

This study aimed to evaluate the clinical parameters, proliferative index, and BAX and Bcl-2 expression in dogs with cSCC that underwent ECT. To the best of the our knowledge, this is the first study to evaluate the expression of BAX, Bcl-2, and Ki67 in dogs with cSCC that underwent ECT.

## Results

### Clinical data

**Figure 1:**
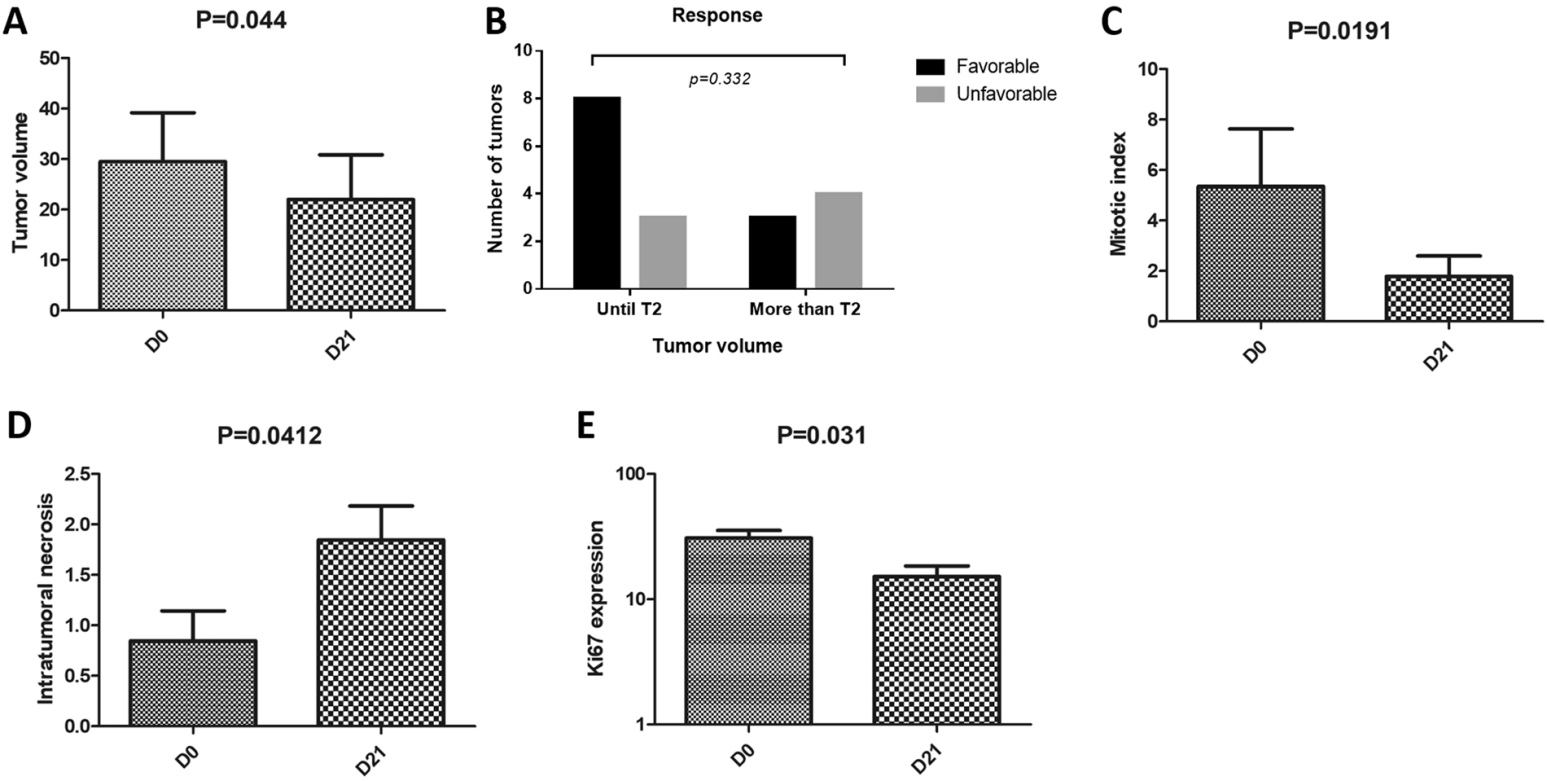
Evaluation of tumor volume, clinical response by tumor volume, mitotic index, intratumoral necrosis and Ki67 expression before and after treatment with ECT in dogs with cSCC. (**A**) The tumor volume before the treatment ranged from 0.14 to 112.9 cm³ (median of 4.64) and significantly decreased after treatment (p = 0.04), ranging from 0.11 to 118.2 cm³ (median of 1.49). (**B**) Evaluation of tumor size at D0 as a prognostic factor based on a cutoff of 5 cm. The volume at D0 had no impact on survival and no prognostic value (P > 0.05), and the response promoted by ECT was not significant (p = 0.332). (**C**) A decreased mitotic index at D21 (median of 1.5, ranging from 0 to 20) (P = 0.019) was observed. (**D**) More intratumoral necrosis was observed in tumor samples at D21 than at D0 (P = 0.041). (**E**) A lower proliferative index (p = 0.031) was observed at D21 than at D0.

Among the 11 subjects, only three had regional lymph node involvement at the time of diagnosis. There was no difference in survival between subjects with and subjects without lymph node involvement (Supplementary Fig. 1A) (P > 0.05). Among the 18 lesions, the tumor volume before treatment ranged from 0.14 to 112.9 cm³ (median of 4.64), and the volume decreased significantly after treatment (p = 0.04), ranging from 0.11 to 118.2 cm³ (median of 1.49) (Supplementary Table 1) (Fig. 1A).

Based on the volume measurements, 11 (61.1%) lesions exhibited partial remission (PR), 3 (16.6%) exhibited stable disease (SD), and 4 (22.2%) exhibited progressive disease (PD). We evaluated the tumor size at D0 as a prognostic factor according to the TNM recommendation for human SCCs, and we grouped the subjects based on tumor volume (cutoff of 5 cm^3^). The volume at D0 had no impact on survival (Fig. 1B) and no prognostic value (P > 0.05).

**Figure 2:**
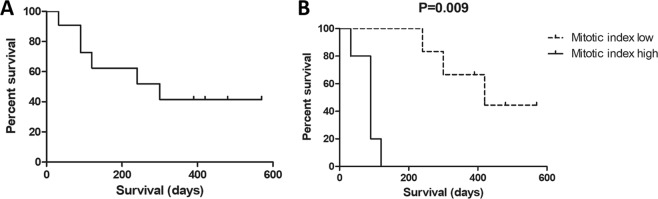
Overall median survival of the 11 dogs and survival based on the mitotic index. (**A**) The median survival of the 11 dogs was 180 days (32 to 570 days). (**B**) Subjects with mitosis numbers lower than 4.9 at D0 survived for a longer time than subjects with mitosis numbers higher than 4.9 at D0 (P = 0.009).

In addition, the association between the clinical stage before treatment (D0) and the response to ECT was not significant (p = 0.332) (Fig. 1B). The median survival time of the 11 dogs was 180 days (32 to 570 days) (Fig. 2A).

Additional clinical results are shown in Supplementary Table 1. The number of sessions that each subject underwent ranged from one to three. Five subjects underwent a surgical procedure after D21 (four due to PD and one due to the owner’s request).

#### Histopathological features

Regarding histopathological grade, 15 lesions (83.3%) were classified as well-differentiated SCC (Supplementary Fig. 2A), and three (16.6%) were classified as poorly differentiated SCC (Supplementary Fig. 2B). Interestingly, only well-differentiated tumors progressed, and all (3/18) the poorly differentiated cSCCs exhibited a PR.

The median mitotic index in the tumor group at D0 was 4.94 (0 to 34), and a relationship between the mitotic index and overall survival was observed. Subjects with mitosis numbers lower than 4.9 at D0 survived for a longer time than did subjects with mitosis numbers higher than 4.9 at D0 (P = 0.009) (Fig. 2B).

Additionally, we observed decreased mitotic index values at D21 compared to those at D0 (median of 1.5, ranging from 0 to 20) (P = 0.019) (Fig. 1C). We also assessed intratumoral necrosis in the tumor samples at D0 and D21. Not surprisingly, we found more necrosis in tumors at D21 than at D0 (P = 0.041) (Fig. 1D). We evaluated necrosis at D0 and the survival time, and we did not find any significant differences (P > 0.05).

#### Protein expression

**Figure 3:**
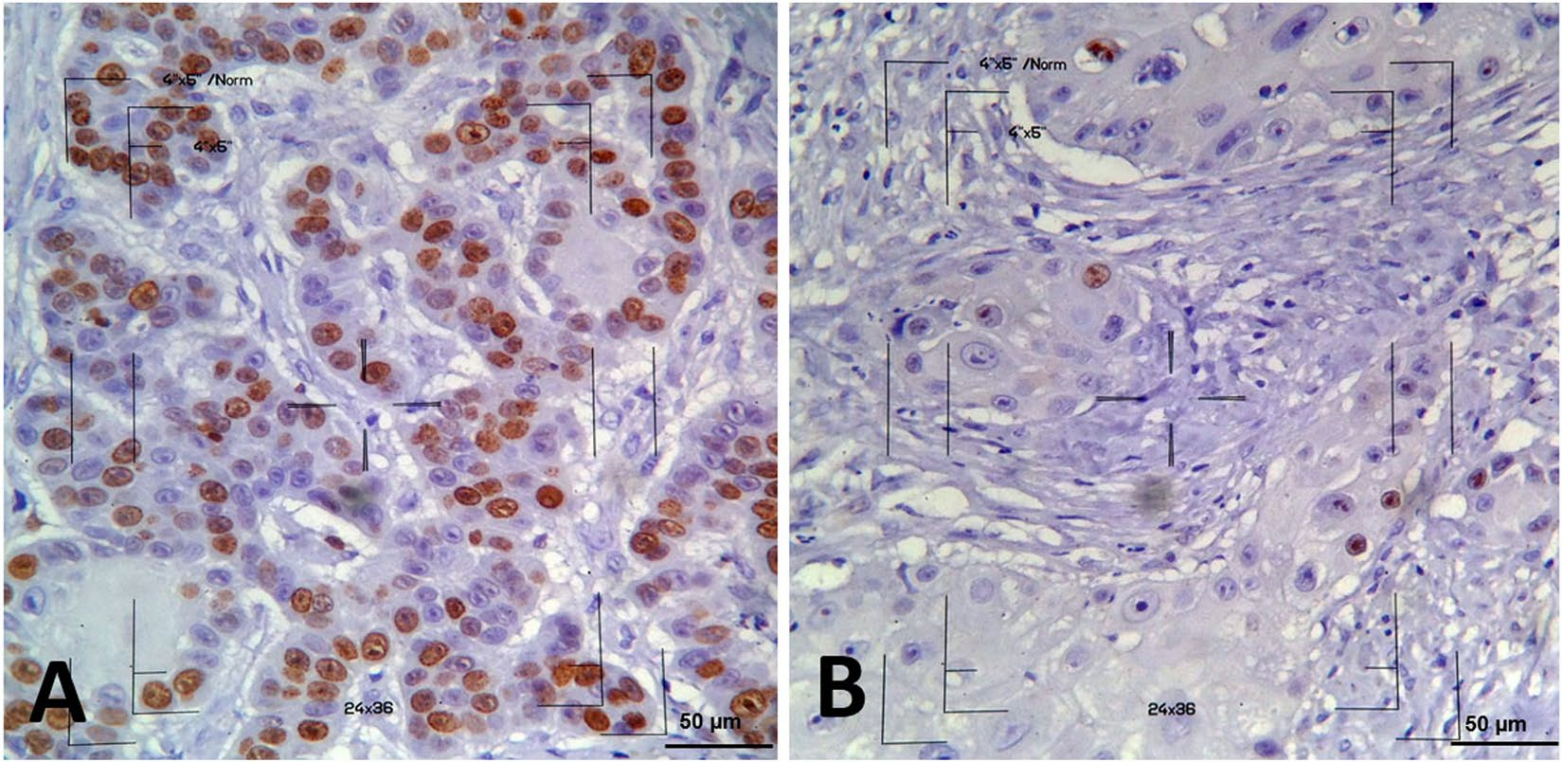
Microscopy images of Ki67 immunostaining in cSCC samples from dogs. (**A**) High nuclear immunostaining of neoplastic cells at D0 (score of 3). (**B**) Low nuclear immunostaining at D21 (score of 1). The 4“x5” reticule used to count the cells is shown in the images (immunocytochemistry, Envision, DAB, counterstaining with Harris hematoxylin, 400×).

Immunohistochemical expression was evaluated in 15 tumors (15/18) at two different time points (D0 and D21); the remaining 3 samples were excluded due to the absence of neoplasticity in tumor samples at D0 or D21. In the remaining samples, the median expression of Ki67 was 277.96 (113.4 to 511.4) at D0 and 193.92 (15 to 494) at D21. We found that the expression of proliferative markers decreased between D0 and D21 (Fig. 3A,B). Thus, tumor samples after ECT (D21) had a lower proliferative index than those before ECT (p = 0.031) (Fig. 1E). Using the proliferative index at D0, we evaluated the survival of subjects with Ki67 values lower and higher than the median Ki67 value, and we did not find a significant difference (P > 0.05).

**Figure 4:**
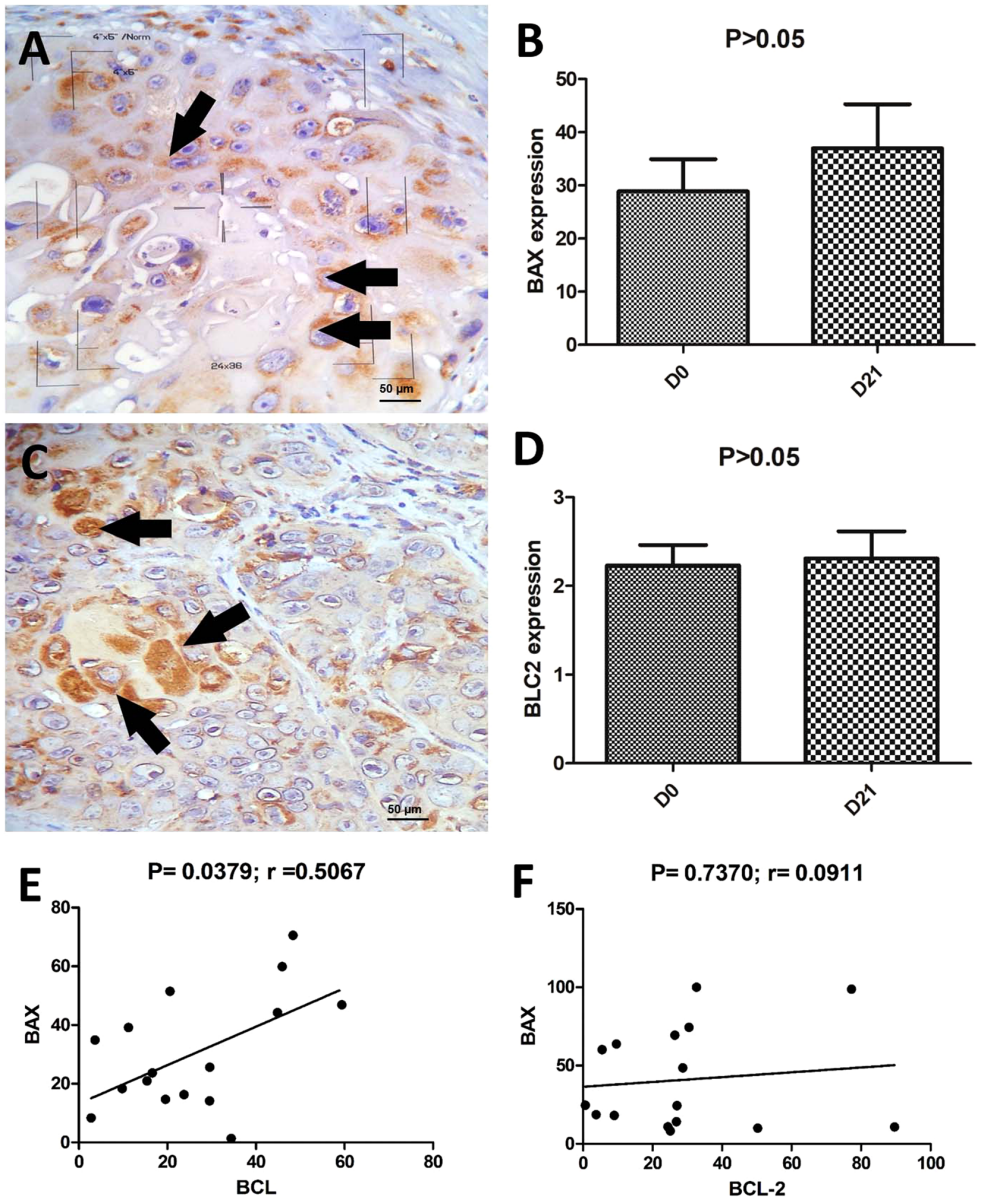
BAX and Bcl-2 immunostaining and correlation of both apoptotic markers with cSCC before and after ECT. (**A**) Cytoplasmic expression of BAX (arrows). (**B**) There was no significant difference in BAX expression between D0 and D21 (P > 0.05). (**C**) Cytoplasmic expression of Bcl-2 (arrows). (**D**) There was no statistical significance of Bcl-2 expression between D0 and D21 (P > 0.05). (**E**) Positive correlation between BAX and Bcl-2 expression before ECT (D0) (p = 0.0379, r = 0.5067) (**F**) Twenty-one days after treatment, no significant Gaussian approximation was observed (p = 0.7370, r = 0.0911).

Both BAX and Bcl-2 protein was expressed in the cytoplasm (Fig. 4A). The median BAX expression level was 21.02% (ranging from 8.34 to 70.56) at D0 and 24.53% (ranging from 10 to 74.47) at D21, and there was no significant difference in BAX expression between D0 and D21 (P > 0.05) (Fig. 4B). There was also no significant difference in overall survival between subjects with low BAX expression and those with high BAX expression (P > 0.05) (Supplementary Fig. 1C).

The median Bcl-2 expression level was 20.62% (ranging from 2.76% to 48.37) at D0 and 26.42% at D21 (ranging from 0.65 to 77.23) (Fig. 4C). There was no statistically significant difference in Bcl-2 expression between D0 and D21 (P > 0.05) (Fig. 4D). When we evaluated the survival of animals with low and high Bcl-2 expression, we did not find a significant difference (P > 0.05) (Supplementary Fig. 1D).

Furthermore, a positive correlation was observed between BAX and Bcl-2 expression levels before ECT (D0) (p = 0.0379, r = 0.5067) (Fig. 4E). However, after treatment (D21), we did not find a significant Gaussian approximation (p = 0.7370, r = 0.0911) (Fig. 4F). Regarding the correlation between BAX and Ki67 and between Bcl-2 and Ki67, no significant difference was observed at any of the time points (p > 0.05).

### Gene expression

The mean relative quantification (RQ) of BAX expression was 1.52 ± 0.48 at D0 and 1.46 ± 0.35 at D21 and of Bcl-2 expression was 1.35 ± 0.45 at D0 and 1.33 ± 0.34 at D21. There was no correlation between BAX and Bcl-2 gene expression at either D0 (Spearman R = 0.1826; p = 0.5478) or D21 (Spearman R = 0.0876; p = 0.7748).

## Discussion

cSCC is a very common tumor subtype in tropical regions, and one of the largest therapeutic challenges in these regions is reducing or eliminating sun exposure during treatment. Because cSCC has a low-level response to chemotherapy and a low metastatic rate, a localized approach is required to achieve long-term disease-free intervals and overall survival. Surgery is the primary treatment for cSCC; however, many patients have multiple tumors at the time of diagnosis, which makes surgical approaches difficult. Therefore, new techniques are required to improve the outcomes of canine patients with cSCC.

In this study, we evaluated ECT as a primary therapy for cSCC and determined the association of different parameters with overall survival and tumor response to ECT. We observed a reduction in tumor size after ECT (D21), even in subjects with poorly differentiated tumors. Four lesions (in four different subjects) exhibited PD. Of these four lesions, two had large volumes (ID 5 and 8), and tumors with a large volume may have a more limited response, as neither homogenous intratumoral concentrations of bleomycin and doxorubicin nor homogeneity of electric pulses may be effective. Unfortunately, no veterinary medicine studies have indicated an association of a tumor size cutoff with prognosis. In a study on the use of ECT for basal cell carcinoma (BCC) in a large series of humans, a 50% complete remission (CR) rate was observed after a single ECT cycle in patients with primary BCC. Interestingly, in the same study, a second ECT cycle increased the CR rate from 50 to 63%, and retreatment was more advantageous in patients with local BCC. Thus, retreatment with ECT seems to be a reasonable option for reducing the response duration in patients with small BCC tumors^52^. In accordance with that human study, our results indicated that ECT could be a good therapeutic option for small tumors, whereas large tumors may need more than one round of ECT. A previous case report published by our research group reported CR of digital trichoblastoma after three sessions of ECT, reaffirming the usefulness of repeated ECT sessions for larger tumors^20^. Another study also evaluated the response of SCC at the second month post-ECT, observing 55% of patients achieving CR, (43% for tumors less than 3 cm and 12% for tumors larger than 3 cm)^53^. Furthermore, Matthiessen *et al*.^54^ also observed a correlation between tumor size and response to ECT, as we have reported in this study.

Among the factors limiting our study, there was no cutoff point for tumor size in cutaneous neoplasms in dogs, unlike in humans. In addition, the staging adopted in veterinary medicine does not consider the size of the subject, which may influence the behaviors of tumors of the same size in dogs of different sizes. The small sample size is also a limitation of our study. However, we performed a G power analysis to ensure that there was a sufficient number of samples in the tumor group.

Interestingly, lymph node involvement was not a prognostic factor in dogs with cSCC. However, we had a small number of subjects with lymph node metastasis, which made it difficult to perform any analyses. According to the literature, the number and size of the lesions seemed to be more important than the involvement of local lymph nodes^1,12,34^. This result can be explained by the invasive behavior of cSCC^1^. Usually, these tumors are more locally invasive than metastatic tumors^1,12,34^. On the other hand, the number of mitotic events was correlated with overall survival in our study. Because the mitotic index is a routine evaluation, it will be important to evaluate the sensitivity and specificity of these indices to predict outcomes in dogs with cSCC; however, a large sample size is necessary. We also found more necrosis in samples after ECT, indicating that this therapy induces both apoptosis and necrosis. Thus, the higher amount of necrosis observed at D21 compared to that at D0 was due to the therapy.

One of the most interesting results of our study was the decreased proliferative index of tumors after ECT compared to that of tumors before ECT. Because ECT induces tumor apoptosis and necrosis, the number of proliferating cells was reduced. Furthermore, we did not find any difference in apoptosis between the samples at the two time points (D0 and D21), indicating that another mechanism may be involved in inhibiting tumor proliferation after ECT. Because ECT is a recent technique in veterinary medicine, there are only a limited number of studies that have demonstrated the antitumoral effect of ECT on cSCC.

Due to established *in vitro* and *in vivo* evidence of the use of doxorubicin in ECT^44,46,47,51^, we included doxorubicin in our study design. However, our results clearly do not support the use of doxorubicin in ECT. We also did not observe any toxic effects of doxorubicin (data not shown) in the subjects studied. Doxorubicin is a more expensive drug and did not show an increased antitumor response in our tumor group compared with the previous literature for canine cSCC, oral carcinoma or digital trichoblastoma, which reported survival times of 600, 923 and 700 days, respectively^20,55,56^. In addition, in evaluating the proapoptotic markers BAX and Bcl-2 in tumor samples after ECT with bleomycin and doxorubicin, we did not find evidence of apoptosis activation. Thus, the use of doxorubicin also did not interfere with apoptosis.

In general, the high Ki67 expression observed in SCC, especially in poorly differentiated SCC, is related to an aggressive phenotype^9,36,39,57^. However, in the lesions studied, the proliferation index was considered high despite the predominance of well-differentiated SCCs (83.3%), suggesting an aggressive phenotype despite the high degree of differentiation. Additionally, there is no established cutoff point for Ki67 expression in dogs that can be considered an indicator of recurrence or metastasis as there is for cutaneous mast cell tumors, which is 23 positive cells in a 10 mm × 10 mm/400 × area^58^. It should be noted that the complete evaluation of proliferation markers may be more effective for predicting tumor biological behavior than the evaluation of a single marker. In this context, Auler *et al*.^57^ reported a case of a dog with well-differentiated SCC in its foreskin with inguinal lymph node metastasis. This animal showed high Ki67 positivity in the tumor tissue as well as high expression levels of the growth factors HER-2 and epidermal growth factor receptor (EGFR).

A previous study observed the same proliferative index pattern in mast cell tumors as that shown in this study, reporting fewer cells that were positive for Ki67 among neoplastic cells 28 days after treatment^19^.

Regarding the apoptotic markers, all cSCC lesions showed cytoplasmic expression of BAX protein. These results differ from those of Madewell *et al*.^30^, who observed less than 2% BAX expression in cSCC in felines. However, other human studies observed BAX expression in cSCC and pulmonary SCC, which ranged from 53–100%^59,60^. A recent study reported that BAX expression in normal keratinocytes in the dog epidermis ranged from very weak to moderate, highlighting the importance of this expression in the pathogenesis of diseases^61^.

Likewise, no significant difference in the expression of the antiapoptotic Bcl-2 protein was observed before and after treatment with ECT. This is the first study to evaluate whether the immunohistochemical expression of the Bcl-2 protein is altered by ECT in cSCC. A previous report evaluated Bcl-2 expression in mast cell tumors and observed increased levels of anti-Bcl-2 at D28 after ECT and then decreased expression at D46^19^. In our study, all the cSCC samples showed immunolabeling of Bcl-2. However, other studies reported different results, and only one study used immunohistochemistry to evaluate Bcl-2 expression in dogs with cSCC (n = 5), among which only one dog showed positivity^62^. In humans, Puizina-Ivic *et al*.^63^ observed an absence of Bcl-2 positivity in SCC (n = 20), whereas Abu Juba *et al*.^55^ observed positivity in 50% of cSCC samples (n = 10).

The correlation between BAX and Bcl-2 expression was significant before treatment, but no difference was observed afterwards; moreover, no difference was observed between either BAX and Ki67 or Bcl-2 and Ki67 at either time point. In human studies, the BAX/Bcl-2 ratio can act as a rheostat that determines cell susceptibility to apoptosis^64^, and lower levels of this ratio may lead to resistance of human cancer cells to apoptosis. In colorectal tumors, BAX and Bcl-2 expression levels were the most predictive of patient outcomes when the BAX/Bcl-2 expression ratio was recorded^65^.

Although the chemotherapeutic agents used have the capacity to damage cells and induce cell death by apoptosis, the BAX and Bcl-2 markers in the samples were not altered at the time point evaluated (D21). It is possible that other proteins involved in the apoptosis pathways exert a greater influence on ECT-mediated cell death. In addition, it cannot be ruled out that, in view of the dynamic interplay and complexity of the apoptosis process, BAX and Bcl-2 expression may have been affected by ECT prior to but not at D21. A limitation in scoring a single protein is that monitoring a single protein may not reflect the level of apoptosis owing to the multifaceted and dynamic nature of this process.

Regarding the proliferative index, 21 days after application of ECT, significant reductions in the proliferative indices were observed in the lesions sampled. The same trend was also observed in mammary neoplasms from human patients subjected to systemic chemotherapy, as a significant reduction in the Ki67 expression in the tumor was observed at 21 days of treatment^66,67^.

ECT was able to reduce the tumor volume and cellular proliferation of cSCC. Furthermore, the gene and protein expression levels of BAX and Bcl-2 were not altered in response to ECT at the time points evaluated. Therefore, studies that serially evaluate these proteins, as well as investigate other proteins involved in apoptosis, may further our understanding of the effect of ECT on apoptosis in canine cSCC.

## Materials and Methods

### Ethical approval

This study was performed in accordance with the National and International Recommendations for the Care and Use of Animals (National Research Council)^68^. All procedures were performed after receiving approval from the Ethics Committee on Animal Use (CEUA) of Veterinary Teaching Hospital of University of Franca (CEUA/UNIFRAN, #033/15).

### Study design

A prospective nonrandomized clinical study was performed using dogs that presented to the Veterinary Teaching Hospital of São Paulo State University (UNESP) and the Veterinary Teaching Hospital of University of Franca (UNIFRAN) with naturally occurring cSCC and that were treated with ECT. Consent to perform the treatment was obtained from the dogs’ owners. The recommendations made by Campana *et al*.^69^ for reporting clinical studies on ECT were followed. Follow-up was defined from initial enrollment in the study until the last examination of the subject, with a minimum of one month.

### Inclusion criteria and animal selection

All dogs enrolled in this study fulfilled the following criteria: histopathologically confirmed diagnosis of stage T1 cSCC (according to the World Health Organization)^70^; absence of distant metastases; compliance of the owner with follow-up after 21 days; and owner’s permission to perform biopsies before (D0) and after (D21) ECT.

Mixed breed dogs (n = 5) were the dogs most commonly affected by cSCC, followed by American Pit Bulls (n = 3), Boxers (n = 2) and English Pointers (n = 1). All subjects had sparse fur and lightly pigmented skin. The mean age of the animals was 7.5 ( ± 2.29) years, with females being the more common sex (n = 10). Five animals had more than one lesion; thus, we investigated 18 lesions in 11 subjects.

The most commonly affected sites observed were the abdominal region (n = 10) followed by the thoracic region (n = 5), axillar region (n = 1), preputial region (n = 1) and tibial region (n = 1). The inclusion criteria were as follows: clinically staged cSCC and records of complete physical examinations, laboratory exams, and fine needle aspiration of regional lymph nodes. Three-view thoracic radiography and abdominal ultrasonography were also performed.

### Electrochemotherapy protocol

Bleomycin (Cinaleo®-Meizler, Barueri-SP) was diluted in 5 mL of saline solution and administered intravenously (IV) at a dose of 15,000 UI/m^2^. Five minutes after bleomycin administration, doxorubicin (Cloridrato de Doxorrubicina® - Eurofarma Laboratórios S.A. Ribeirao Preto – SP) was diluted in 25 mL of saline solution and administered IV at a dose of 30 mg/m², followed by a sequence of 8 biphasic electric pulses, each lasting 100 ms. The 1000 V pulses, administered at a frequency of 1 Hz, were generated by portable electroporator (LC BK-100, Brazil) and delivered by six needle electrodes with a 0.3 mm distance between them; the needle electrodes were arranged in rows (parallel array) until they completely covered the tumor. The procedure lasted 28 minutes as described by a previous report^71^. Treatment was repeated when macroscopic lesions were still observed 21 days after the procedure. The 21-day interval treatment was based on the doxorubicin protocol.

All ECT procedures were administered under general anesthesia induced using IV-administered propofol (Provive®, União Química - Farmacêutica Nacional S/A. São Paulo, SP, Brazil) (5 mg/kg) followed by endotracheal intubation; anesthesia was maintained with isoflurane (Isoforine®, Cristália Produtos Químicos Farmacêuticos Ltda. Itapira, SP, Brazil). All animals received postoperative analgesia, including an IV injection of meloxicam (0.2 mg/kg) (Maxicam 0.2%®, OuroFino, São Paulo, SP, Brazil) and tramadol (2 mg/kg) (Tramal® União Química - Farmacêutica Nacional S/A, São Paulo, SP, Brazil).

### Tumor response

The total neoplasm volume was calculated by the following formula: π × length × width × height/6; the tumor volume was measured at D0 and D21 using a digital pachymeter^72^. The same evaluator measured tumor volume to prevent measurement bias. A CR was defined as a total reduction in the measured tumor volume, while a PR was defined as a ≥ 30% reduction in tumor volume. SD was defined as a ˂ 30% reduction in tumor volume or a ˂ 20% increase in tumor volume, and PD was defined as a ≥ 20% increase in tumor volume or the presence of new lesions^73^. A CR or PR was considered a “favorable” response, while PD and SD were considered “unfavorable” responses. In our study design, we evaluated tumor response at a 21-day interval (3 weeks) instead of at 4 weeks based on the doxorubicin protocol.

### Histopathological evaluation

The first sample was collected immediately before the first ECT session (D0), and the second sample was obtained on day 21 (D21) after ECT. A 6-mm punch biopsy was used to obtain the tumor samples. All samples were immediately placed in 10% formalin for 24 hours, followed by 70% alcohol until the paraffinized sections were prepared. Afterwards, hematoxylin and eosin staining was performed as previously described^74^. All tumor samples were classified according to the criteria established by Gross *et al*.^5^ as follows: 1) well-differentiated SCC presenting centralized accumulation of compact laminated keratin or keratin pearls, with keratinization progressing through the granular cell layer similar to the normal epidermis and the keratinized centers of the lobules undergoing necrosis and becoming infiltrated by neutrophils, or 2) poorly differentiated SCC presenting smaller epithelial structures, cords and nests rather than large islands of squamous epithelial cells, moderate to high mitotic activity, and no keratin pearls. In addition, Broder’s grading system was also included, which characterizes well-differentiated SCC as grade 1, moderately differentiated as grade 2 and 3 and poorly differentiated as grade 4^75^. The mitotic index was established by counting the number of mitotic cells in relation to the total number of cells in an ocular grid per five random high-power fields (400×).

### Immunohistochemistry

Immunohistochemical staining with BAX, BCL-2 and Ki67 antibodies was performed in all cSCC tissues using the original biopsy samples (D0) and the biopsy specimens collected 21 days after the first ECT (D21). Immunohistochemical staining was performed using the peroxidase method and 3,3’ diaminobenzidine tetrachloride (DAB). The slides were dewaxed in xylol and rehydrated in graded ethanol. For antigen retrieval, the slides were incubated in citrate buffer (pH 6.0) in a pressure cooker (Pascal, Agilent Technologies, Santa Clara, CA, USA). Endogenous peroxidase was blocked with a commercial solution (Protein Block, Agilent Technologies, Santa Clara, CA, USA), and the samples were incubated with the following primary antibodies overnight at 4 °C: monoclonal anti-BAX (mouse monoclonal, Santa Cruz Biotechnology, Dallas, TX, USA), 1:200 dilution; anti-Bcl-2 (mouse monoclonal, Santa Cruz Biotechnology, Dallas, TX, USA) 1:400 dilution; and anti-Ki67 (MIB-1, Agilent Technologies, Santa Clara, CA, USA), 1:50 dilution. After incubation with the above antibodies, the slides were placed on an autostainer platform (Agilent Technologies, Santa Clara, CA, USA). Then, the sections were counterstained with Harris’s hematoxylin, dehydrated, and mounted. The cross-reactivity of anti-Ki67 antibody with canine tissue was provided by the manufacturer on the antibody datasheet.

Negative controls were run for all antibodies with a universal negative control mouse antibody (Dako, Carpinteria, CA, USA) according to the manufacturer’s instructions. The positive control consisted of normal lymph nodes for all antibodies according to Protein Atlas guidelines (www.proteinatlas.org).

### Immunohistochemical evaluation

The immunohistochemical slides were examined under a light microscope using an ocular grid 26 mm in diameter (Leica Microscopy DMLB, HC, PLAN 10 × /20, 4″ × 5″) at 400 × magnification. Ki67 immunostaining was assessed by counting the number of positive cells inside the ocular grid area. We counted five random high-power fields (400×), and the final result was the median of the five fields for each sample. Regarding BAX and Bcl-2 expression, we counted the number of positive and negative cells in 10 high-power fields. Then, we calculated a score of positive cells by dividing the total number of positive cells by the total number of cells (positive and negative) and expressed the scores as a percentage of positive expression. In evaluating the scores, cytoplasmic staining for BAX and Bcl-2 and nuclear staining for Ki67 were considered.

### Gene expression

We evaluated BAX and Bcl2 gene expression in formalin-fixed, paraffin-embedded tissues from samples collected at D0 and D21. mRNA was extracted using a commercial RecoverAll™ Total Nucleic Acid Kit (Ambion, Life Technologies, MA, USA) according to the manufacturer’s instructions. Determination of the mRNA concentration and integrity as well as cDNA synthesis were performed as previously described^76^. The BAX and Bcl2 primers^77^ as well as the reference gene primers^76^ were designed as previously described and added to a final concentration of 0.3 μM for each primer in a total volume of 10 μL containing Power SYBR Green PCR Master Mix (Applied Biosystems; Foster City, CA, USA), 1 μL of cDNA (1:10). The reactions were performed in triplicate in 384-well plates using QuantStudio 12 K Flex Thermal Cycler equipment (Applied Biosystems; Foster City, CA, USA). A dissociation curve was included in all experiments to determine the PCR product specificity. Relative gene expression was quantified using the 2^−ΔΔCT^ method^78^.

### Statistical analysis

For statistical purposes, we calculated the median value of each clinical, histopathological and immunohistochemical parameter and classified the value for each parameter as “low” when it was lower than the median and “high” when it was greater than the median. Then, we compared the survival rates of dogs with low and high values of each parameter using the Kaplan-Meier method. For the survival curve, subjects that died from other diseases or that were lost to follow-up were censored. When the subjects died from the disease, they were classified as uncensored. To evaluate the association between the tumor stage (≤T2 x > T2) before and after ECT, Fisher’s exact test was applied. Furthermore, Spearman’s correlations of the BAX, Bcl-2 and Ki67 expression levels were used to compare the levels at two time points, namely, before and after ECT. The BAX and Bcl2 gene expression levels were evaluated using the Mann-Whitney test. Commercial software (GraphPad Prism® 8.1.0 – GraphPad Software) was used for the statistical analysis. P values < 0.05 were considered significant.

## Supplementary information

Suppl Fig. 1, 2, table 1

## References

[1] Gross, T., Ihrke, P. J., Walder, E. J. & Affolter, V. K. Epidermal tumors. In Skin diseases of the dog and cat. Clinical and histopathologic diagnosis. Publisher Oxford: Blackwell Science Ltd, pp. 581–585 (2005).

[2] Bevier DF, Goldschmidt MH (1981). Skin tumors in the dog. Part I: Epithelial tumors and tumor-like lesions. Comp. Cont. Ed..

[3] Goldschmidt, M. H. & Shofer, F. S. Skin Tumors of the Dog and Cat. Publisher Pergamon Press, Oxford, pp. 37–49 (1982).

[4] Muller, W. H. M. & Kirk, R. Neoplastic and Non-neoplastic tumors. In Muller Kirk. Small Animal Dermatology, 7th ed.;Publisher Elsevier, St. Louis, Missouri, pp. 774–780 (2013).

[5] Teifke, J.P., Lohr, C.V. & Shirasawa, H. Detection of canine oral papillomavirus- DNA in canine oral squamous cell carcinomas and p53 overexpressing skin papillomas of the dog using the polymerase chain reaction and non-radioactive *in situ* hybridization. Vet. Microbiol. 60, 119–130 (1998). 10.1016/s0378-1135(98)00151-5 9646444

[6] Schwegler K, Walter JH, Rudolph R (1997). Epithelial neoplasms of the skin, the cutaneous mucosa and the transitional epithelium in dogs: an immunolocalization study for papillomavirus antigen. Zentralblatt Vet..

[7] Kirkham N. Tumors and cysts of the epidermis. In Lever’s Histopathology of the Skin, 8th ed.; Publisher Lippincott Raven, Philadelphia, pp. 712–17 (1997).

[8] Mittelbronn MA, Mullins DL, Ramos-Caro FA, Flowers FP (1998). Frequency of pre-existing actinic keratosis in cutaneous squamous cell carcinoma. Int. J. Dermatol..

[9] Poggiani SSC, Hatayde MR, Laufer-Amorim R, Werner J (2012). Expression of Cyclooxygenase-2 and Ki-67 in Actinic Keratosis and Cutaneous Squamous Cell Carcinoma in Dogs. Open J. Vet. Med..

[10] Rogers KS, Helman RG, Walker MA (1995). Squamous cell carcinoma of the canine nasal planum: eight cases (1988-1994). J. Am. Anim. Hosp. Assoc..

[11] Kirpensteijn J, Withrow SJ, Straw RC (1994). Combined resection of the nasal planum and premaxilla in three dogs. Vet. Surg..

[12] Lascelles BD, Parry AT, Stidworthy MF, Dobson JM, White RA (2000). Squamous cell carcinoma of the nasal planum in 17 dogs. Vet. Rec..

[13] Withrow SJ, Straw RC (1990). Resection of the nasal planum in nine cats and five dogs. J. Am. Anim. Hosp. Assoc..

[14] Brougham ND, Dennett ER, Cameron R, Tan ST (2012). The incidence of metastasis from cutaneous squamous cell carcinoma and the impact of its risk factors. J. Surg. Oncol..

[15] Karia PS (2014). Evaluation of American Joint Committee on Cancer, International Union Against Cancer, and Brigham and Women’s Hospital tumor staging for cutaneous squamous cell carcinoma. J. Clin. Oncol..

[16] Schmults CD, Karia PS, Carter JB, Han J, Qureshi AA (2013). Factors predictive of recurrence and death from cutaneous squamous cell carcinoma: a 10-year, single-institution cohort study. JAMA Dermatology..

[17] Spugnini EP, Azzarito T, Fais S, Fanciulli M, Baldi A (2016). Electrochemotherapy as First Line Cancer Treatment: Experiences from Veterinary Medicine in Developing Novel Protocols. Curr. Cancer Drug Targets..

[18] Spugnini EP (2007). A. Patterns of tumor response in canine and feline cancer patients treated with electrochemotherapy: preclinical data for the standardization of this treatment in pets and humans. J. Transl. Med..

[19] Salvadori C (2017). A. Effects of electrochemotherapy with cisplatin and peritumoral IL-12 gene electrotransfer on canine mast cell tumors: a histopathologic and immunohistochemical study. Radiol. Oncol..

[20] Dos Anjos DS, Rossi YA, Magalhaes LF, Calazans SG, Fonseca-Alves CE (2018). Digital trichoblastoma treated with electrochemotherapy in a dog. Vet. Record..

[21] Groeger AM (2004). Prognostic value of immunohistochemical expression of p53, BAX, BCL-2 and BCL-Xl in resected non-small cell lung cancer. Histopathol..

[22] Karam JA (2007). Use of combined apoptosis biomarkers for prediction of bladder cancer recurrence and mortality after radical cystectomy. Lancet Oncol..

[23] Zeestraten ECM (2013). The Prognostic Value of the Apoptosis Pathway in Colorectal Cancer: A Review of the Literature on Biomarkers Identified by Immunohistochemistry. Biomark. Cancer..

[24] De Bruin EC, Medema JP (2008). Apoptosis and non-apoptotic deaths in cancer development and treatment response. Cancer Treat Rev..

[25] Meichner K, Fogle JE, English L, Suter SE (2016). Expression of Apoptosis-regulating Proteins Bcl-2 and Bax in Lymph Node Aspirates from Dogs with Lymphoma. J. Vet. Intern. Med..

[26] Dolka L, Król M, Sapierzynski R (2016). Evaluation of apoptosis-associated protein (Bcl-2, Bax, cleaved caspase-3 and p53) expression in canine mammary tumors: An immunohistochemical and prognostic study. Res. Vet. Sci..

[27] Yildirim F (2014). Evaluation of Bcl-2, Bcl-xl, and Bax expression and apoptotic index in canine mammary tumours. Kafkas Univ. Vet. Fak. Derg..

[28] Sirivisoot S, Teewasutrakul P, Rungsipipat A, Tangkawattana S, Techangamsuwan S (2018). Transcriptome analysis of abcb1, abcg2 and the bcl2/bax ratio in refractory and relapsed canine lymphomas under treatment and rescue protocol. Acta Vet. Beogard..

[29] Beffagna G (2017). Circulating Cell-Free DNA in Dogs with Mammary Tumors: Short and Long Fragments and Integrity Index. Plos One..

[30] Madewell BR, Gandour-Edwards R, Edwards BF, Matthews KR, Griffey SM (2001). Bax/Bcl-2: Cellular modulator of apoptosis in Feline Skin and Basal Cell Tumours. J. Comp. Path..

[31] Scase TJ (2006). Canine mast cell tumors: correlation of apoptosis and proliferation markers with prognosis. J. Vet. Inter. Med..

[32] Bergkvist GT (2011). Expression of epidermal growth factor receptor (EGFR) and Ki67 in feline oral squamous cell carcinomas (FOSCC). Vet. Comp. Oncol..

[33] Pereira RS (2013). Ki-67 labeling in canine perianal glands neoplasms: a novel approach of immunohistological diagnostic and prognostic. BMC Vet. Res..

[34] Brodzki A, Lopuszyñski W, Brodzki P, Tatara M (2014). Diagnostic and prognostic value of cellular proliferation assessment with Ki-67 in dogs suffering from benign and malignant perianal tumors. Folia Biol. (Kraków)..

[35] Peña L, Zarate AINR, Pérez-Alenza MD, Cuesta PL, Castaño M (1998). Immunohistochemical Detection of Ki-67 and PCNA in Canine Mammary Tumors: Relationship to Clinical and Pathologic Variables. J. Vet. Diagn. Invest..

[36] Khodaeiani E (2013). Immunohistochemical Evaluation of p53 and Ki67 expression in Skin Epithelial Tumors. Indian J. Dermatol..

[37] Xie S (2016). What is the Prognostic Significance of Ki-67 Positivity in Oral Squamous Cell Carcinoma?. J. Cancer..

[38] Batinac T (2006). Expression of cell cycle and apoptosis regulatory proteins in keratoacanthoma and squamous cell carcinoma. Pathol. Res. Pract..

[39] Stratigos AJ, Kapranos N, Petrakou E, Anastasiadou A, Pagouni A, Christofidou E, Petridis A, Papadopoulos O, Kokka E, Antoniou C, Georgala S, Katsambas AD (2005). Immunophenotypic analysis of the p53 gene in non-melanoma skin cancer and correlation with apoptosis and cell proliferation. Journal of the European Academy of Dermatology and Venereology.

[40] Cadossi R, Ronchetti M, Cadossi M (2014). Locally enhanced chemotherapy by electroporation: Clinical experiences and perspective of use of electrochemotherapy. Future Oncol..

[41] Spugnini EP, Baldi A (2014). Electrochemotherapy in veterinary oncology: From rescue to first line therapy. Methods Mol. Biol..

[42] Gehl J, Skovsgaard T, Mir LM (1998). Enhancement of cytotoxicity by electropermeabilization: an improved method for screening drugs. Anticancer Drugs..

[43] Yanai H, Kubota Y, Nakada T (2002). Effects of electropermeabilization after the administration of anticancer drugs on transitional cell carcinoma. BJU Int..

[44] Shil P, Kumar A, Vidyasagar PB, Mishra KP (2006). Electroporation enhances radiation and doxorubicin-induced toxicity in solid tumor *in vivo*. J Environ Pathol Toxicol Oncol..

[45] Ogihara M, Yamaguchi O (2000). Potentiation of effects of anticancer agents by local electric pulses in murine bladder cancer. Urol Res..

[46] Meschini S (2012). Electroporation adopting trains of biphasic pulses enhances *in vitro* and *in vivo* the cytotoxic effect of doxorubicin on multidrug resistant colon adenocarcinoma cells (LoVo). European J Cancer..

[47] Kulbacka J (2014). Doxorubicin delivery enhanced by electroporation to gastrointestinal adenocarcinoma cells with P-gp overexpression. Bioelectrochemistry..

[48] Kulbacka J (2010). Oxidative alterations induced *in vitro* by the photodynamic reaction in doxorubicin-sensitive (LoVo) and -resistant (LoVoDX) colon adenocarcinoma cells. Exp. Biol. Med..

[49] Ye S, MacEachran DP, Hamilton JW, O’Toole GA, Stanton BA (2008). Chemotoxicity of doxorubicin and surface expression of P-glycoprotein (MDR1) is regulated by the Pseudomonas aeruginosa toxin Cif. Am. J. Physiol. Cell Physiol..

[50] Shen F (2008). Quantitation of doxorubicin uptake, efflux, and modulation of multidrug resistance (MDR) in MDR human cancer cells. J. Pharmacol. Exp. Ther..

[51] Kambe M (1997). Enhancement of the efficacy of anticancer drugs with electroporation: successful electrochemotherapy against gastric cancer cell lines *in vivo* and *in vitro*. Int. J. Clin. Oncol..

[52] Campana LG (2017). Basal cell carcinoma: 10-year experience with electrochemotherapy. J. Transl. Med..

[53] Bertino G (2016). European Research on Electrochemotherapy in Head and Neck Cancer (EURECA) project: Results of the treatment of skin cancer. Eur. J. Cancer..

[54] Matthiessen LW (2011). Management of cutaneous metastases using electrochemotherapy. Acta Oncol..

[55] Boncea AM, Cristea A, Bourdeau P (2019). Electrochemotherapy as treatment for generalised squamous cell carcinoma in a dog. Vet. Record Case Report..

[56] Petra, S. *et al*. Treatment of canine oral squamous cell carcinoma using electrochemotherapy. a case series report, https://arpi.unipi.it/handle/11568/934600?mode=full.451#.XXbFkChKjIU (2018).

[57] Auler PA, Gamba CO, Horta RS, Lavalle GE, Cassali GD (2014). Metastatic well differentiated squamous cell carcinoma in the prepuce of a dog: a report of clinicopathological, immunophenotypic and therapeutic approach. Arq. Bras. Med. Vet. Zootec..

[58] Webster JD, Yuzbasiyan-Gurkan V, Miller RA, Kaneene JB, Kiupel M (2007). Cellular proliferation in canine cutaneous mast cell tumors: Associations with c -KIT and its role in prognostication. Vet. Pathol..

[59] Abu Juba B (2013). Apoptotic markers in photoinduced cutaneous carcinoma. Rom. J. Morphol. Embryol..

[60] Porebska I, Kosacka M, Sobanska E, Wyrodek E, Jankowska R (2015). Comparative expression of apoptotic markers in lung adenocarcinoma and squamous cell carcinoma. Adv. Exp. Med. Biol..

[61] Croci M, Dettwiler M, Vaughan L, Guscetti F (2013). Immunohistochemical expression of Bax and Bak in canine non-neoplastic tissues. Vet. J..

[62] Pieper JB, Stern AW, LeClerc SM, Campbell KL (2015). Coordinate expression of cytokeratins 7 and 14, vimentin, and Bcl-2 in canine cutaneous epithelial tumors and cysts. J. Vet. Diagnost. Invest..

[63] Puizina-Ivic N, Sapunar D, Marasovic D, Miric L (2008). An overview of Bcl-2 expression in histopathological variants of basal cell carcinoma, squamous cell carcinoma, actinic keratosis and seborrheic keratosis. Coll. Antropol..

[64] Raisova M (2001). The Bax/Bcl-2 ratio determines the susceptibility of human melanoma cells to CD95/Fas-mediated apoptosis. J. Invest. Dermatol..

[65] Khodapasand E, Farrokhi F, Kamalidehghan B, Houshmand M (2015). Is Bax/Bcl-2 Ratio Considered as a Prognostic Marker with Age and Tumor Location in Colorectal Cancer?. Iran Biomed. J..

[66] Burcombe R (2006). Evaluation of Ki-67 proliferation and apoptotic index before, during and after neoadjuvant chemotherapy for primary breast cancer. Breast Cancer Res..

[67] Jin G (2015). Evaluation of biomarker changes after administration of various neoadjuvant in breast cancer. Int. J. Clin. Exp. Pathol..

[68] National Research Council. Guide for the Care and Use of Laboratory Animals. Washington, National Academies Press, 8th edition (2011).

[69] Campana LG (2016). Recommendations for improving the quality of reporting clinical electrochemotherapy studies based on qualitative systematic review. Radiol. Oncol..

[70] Owen, L. N. TNM Classification of tumor in domestic animals, World Health Organization (1980).

[71] Gehl J (2018). Update standard operating procedures for electrochemotherapy of cutaneous tumours and skin metastases. Acta Oncol..

[72] Tozon N (2016). Operating Procedures of the Electrochemotherapy for Treatment of Tumor in Dogs and Cats. J Vis Exp..

[73] Eisenhauer EA (2009). New response evaluation criteria in solid tumours: Revised RECIST guideline (version 1.1). Eur. J. Cancer..

[74] Fischer A. H., Jacobson K. A., Rose J., Zeller R. (2008). Hematoxylin and Eosin Staining of Tissue and Cell Sections. Cold Spring Harbor Protocols.

[75] Goldschmidt, M. H. & Goldschmidt, M. H. Epithelial and Melanocytic Tumors of the skin. In: Meuten, D. J. Tumors in Domestic Animal. 5th edition, Wiley Blackwell, p. 80 (2017).

[76] Rivera-Calderón LG (2016). Alterations in PTEN, MDM2, TP53 and AR protein and gene expression are associated with canine prostate carcinogenesis. Res. Vet. Sci..

[77] Meichner K, Fogle JE, English L, Suter SE (2016). Expression of Apoptosis-regulating Proteins Bcl-2 and Bax in Lymph Node Aspirates from Dogs with Lymphoma. J. Vet. Intern. Med..

[78] Livak KJ, Schmittgen TD (2001). Analysis of relative gene expression data using real-time quantitative PCR and the 2(-Delta Delta C(T)) Method. Methods..

