# Supplementary material for: Electrochemotherapy induces tumor regression and decreases the proliferative index in canine cutaneous squamous cell carcinoma
Source: Sci Rep. 2019 Nov 1;9:15819. doi: 10.1038/s41598-019-52461-6 (PMC6825193; doi:10.1038/s41598-019-52461-6)
Supplement: Supplementary file 1 — Suppl Fig. 1, 2, table 1 [file 41598_2019_52461_MOESM1_ESM.pdf]

Electrochemotherapy induces tumor regression and decreases the proliferative index in canine cutaneous squamous cell carcinoma

Denner S. Dos Anjos[1*], Cynthia Bueno[2], Larissa F. Magalhães[3], Georgia M. Magalhães[4], Ewaldo Mattos-Junior[1], Marcela M.R. Pinto[5], Andrigo B. De Nardi[2], Carlos H.M. Brunner[6], Antonio F. Leis-Filho[7], Sabryna G. Calazans[1†], Carlos E. Fonseca-Alves[7,8†*]

[1] Veterinary Science Graduate Program, University of Franca (UNIFRAN), Franca, Brazil

[2] Department of Veterinary Clinic and Surgery, São Paulo State University (UNESP), Jaboticabal, Brazil

[3] Department of Veterinary Pathology, University of Franca (UNIFRAN), Franca, Brazil

[4] Federal Institute of Education, Science and Technology of the South of Minas Gerais - Muzambinho, Minas Gerais, Brazil

[5] Veterinary Pathologist at the CEVEPAT Laboratory, Botucatu, SP

[6] Department of Veterinary Clinic of University Paulista, São Paulo, Brazil

[7] Department of Veterinary Clinic, School of Veterinary Medicine and Animal Science, São Paulo State University (UNESP), Botucatu, São Paulo, Brazil

[8] Institute of Health Sciences, Universidade Paulista – UNIP, Bauru, SP, Brazil.

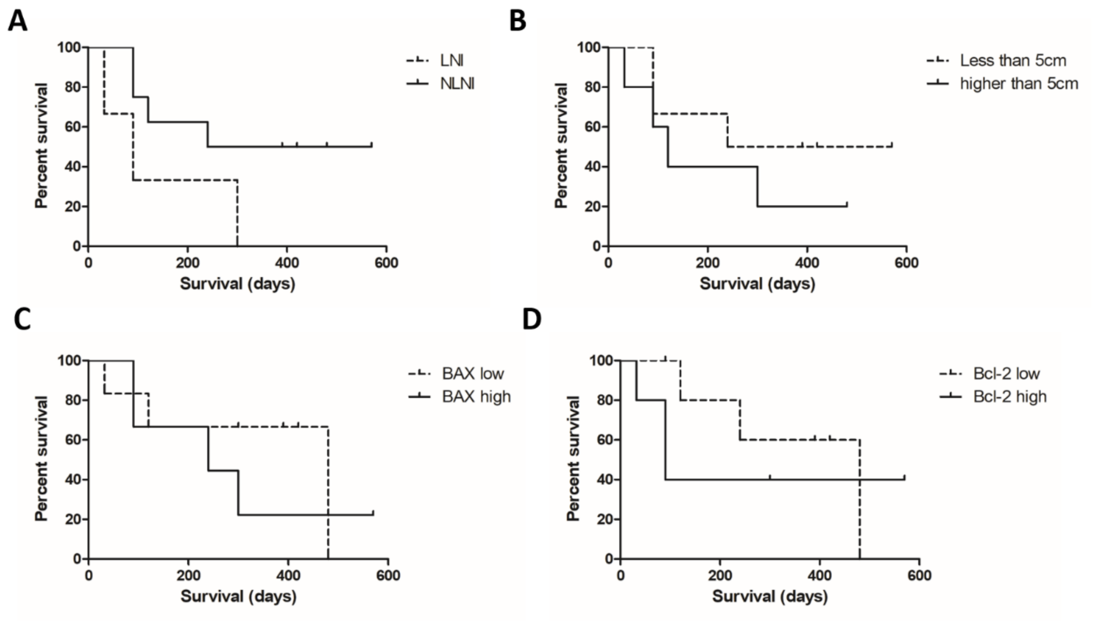

Suppl Fig. 1. Survival analysis according to clinical parameters and immunohistochemistry expression. (A) There was no difference in survival time analysis between subjects with and without lymph node involvements (P > 0,05). (B) There was no statistical different in overall survival of patients according to the tumor size. There was no statistical difference in overall survival between subjects when comparing BAX (C) or Bcl-2 (D) low versus high than the medium value (P>0.05).

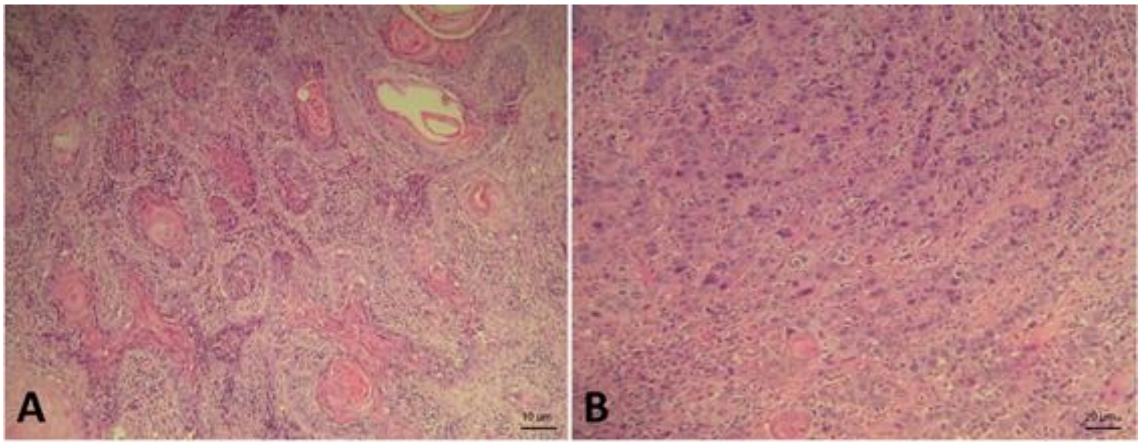

Suppl Fig 2. Histopathological evaluation of squamous cell carcinoma (SCC) in dogs submitted to electrochemotherapy (ECT). (A) Well-differentiated SCC with presence of mononuclear inflammatory infiltrate before ECT (B) SCC before ECT. It is possible o note a tissue disorganization with presence of apoptotic cells showing basophilic nucleus.

Suppl Table 1. Clinical and demographic characteristics of subjects that underwent ECT.

ID	Breed	Age	Sex	Number of tumors	Tumor localization	Histopathological evaluation*	TNM	Tumor size at D0 (cm³)	Tumor size at D21 (cm³)	Outcome	Number of sessions
**1**	Pitbull	8	M	2 (a, b)	Axillar (a)	Poorly differentiated (grade 4)	T2N0M0	3.75	0.91	PR	3 / S
					Preputial (b)	Well differentiated (grade 1)	T2N0M0	19.23	6.44	PR	
**2**	Pitbull	9	F	1	Abdominal	Well differentiated (grade 1)	T2N0M0	5.04	0.73	PR	2
**3**	Boxer	2	F	1	Thorax lateral	Well differentiated (grade 1)	T3N0M0	68.49	65.94	SD	2 / S
**4**	Mixed	10	F	2 (a. b)	Thorax lateral (a)	Poorly differentiated (grade 4)	T2N0M0	2.3	0.278	PR	1 / S
					Abdominal (b)	Well differentiated (grade 1)	T3N0M0	14.15	1.68	PR	
**5**	Mixed	7	F	1	Abdominal	Well differentiated (grade 2 and 3)	T3N0M0	93.57	118.24	PD	2
**6**	Pitbull	9	F	4 (a, b, c, d)	Thorax lateral (a)	Well differentiated (grade 2 and 3)	T1N0M0	1.14	0.175	PR	2 / S

					Abdominal (b)	Poorly differentiated (grade 4)	T3N0M0	112.92	48.2	PR	
					Abdominal (c)	Well differentiated (grade 2 and 3)	T1N0M0	0.81	0.533	PR	
					Abdominal (d)	Well differentiated (grade 2 and 3)	T2N0M0	22.36	5.014	PR	
**7**	Mixed	NA	F	1	Abdominal	Well differentiated (grade 1)	T2N1M0	104.6	104.6	SD	2
**8**	Mixed	NA	F	2 (a. b)	Abdominal (a)	Well differentiated (grade 1)	T3N0M0	4.24	10.36	PD	2
					Thorax lateral (b)	Well differentiated (grade 1)	T2N0M0	0.22	1.046	PD	
**9**	English Pointers	8	F	1	Abdominal	Well differentiated (grade 2 and 3)	T3N1M0	76.93	30.03	PR	2 / S
**10**	Boxer	8	F	2 (a. b)	Thorax lateral (a)	Well differentiated (grade 1)	T1N0M0	0.314	0.113	PR	2
					Tibial (b)	Well differentiated (grade 1)	T1N0M0	0.141	0.28	PD	
**11**	Mixed	7	F	1	Abdominal	Well differentiated (grade 1)	T4N1M0	1.2	1.31	SD	1

M, male; F, female

PR: partial remission; SD: stable disease; PD: progressive disease

S: surgery

NR: death not related to cancer

NF: no follow-up

NA: not available

*Gross and Broder’s grade system
